# Insight into the AcrAB-TolC Complex Assembly Process Learned from Competition Studies

**DOI:** 10.3390/antibiotics10070830

**Published:** 2021-07-08

**Authors:** Prasangi Rajapaksha, Isoiza Ojo, Ling Yang, Ankit Pandeya, Thilini Abeywansha, Yinan Wei

**Affiliations:** Department of Chemistry, University of Kentucky, Lexington, KY 40506, USA; prasangi_iro@uky.edu (P.R.); Isoiza.ojo@uky.edu (I.O.); ling.yang@uky.edu (L.Y.); pandeya.ankit@uky.edu (A.P.); thilini.abeywansha@uky.edu (T.A.)

**Keywords:** RND pump, dominant negative effect, assembly, protein-protein interaction, mutation

## Abstract

The RND family efflux pump AcrAB-TolC in *E. coli* and its homologs in other Gram-negative bacteria are major players in conferring multidrug resistance to the cells. While the structure of the pump complex has been elucidated with ever-increasing resolution through crystallography and Cryo-EM efforts, the dynamic assembly process remains poorly understood. Here, we tested the effect of overexpressing functionally defective pump components in wild type *E. coli* cells to probe the pump assembly process. Incorporation of a defective component is expected to reduce the efflux efficiency of the complex, leading to the so called “dominant negative” effect. Being one of the most intensively studied bacterial multidrug efflux pumps, many AcrA and AcrB mutations have been reported that disrupt efflux through different mechanisms. We examined five groups of AcrB and AcrA mutants, defective in different aspects of assembly and substrate efflux. We found that none of them demonstrated the expected dominant negative effect, even when expressed at concentrations many folds higher than their genomic counterpart. The assembly of the AcrAB-TolC complex appears to have a proof-read mechanism that effectively eliminated the formation of futile pump complex.

## 1. Introduction

Antimicrobial resistance, especially multidrug resistance in bacterial pathogens, is among the top 10 global threats to humanity [1]. Among the large array of different defense mechanisms adapted by bacteria, the overexpression of efflux pumps has a significant role in conferring multidrug resistance. AcrAB-TolC is one of the most extensively studied efflux pump systems in Gram-negative bacteria, playing a crucial role in the multidrug resistance in bacteria such as *Eshcherichia coli* [2,3,4]. AcrAB-TolC is a member of the resistance nodulation division (RND) superfamily. AcrAB-TolC efflux pump confers resistance to a broad spectrum of antimicrobial compounds including β-lactams, tetracycline, novobiocin, and fluroquinolones [5,6]. This tripartite efflux transporter consists of three major protein components [7,8], an outer membrane channel TolC, a periplasmic adaptor protein (PAP) AcrA, and an inner membrane proton-driven antiporter AcrB [9,10,11,12]. TolC forms a channel that spans the outer membrane and acts as the exit pathway of substrates translocated from the inner membrane and the periplasmic space. AcrA has function in stabilizing the connection between the two membrane components TolC and AcrB [13,14]. The RND transporter protein AcrB is responsible for substrate recognition and energy transduction. Upon binding of a substrate, AcrB uses the energy from the proton flow down its concentration gradient through a proton translocation pathway in the transmembrane domain to drive the conformational change necessary to move the substrate upward toward the exit tunnel [4,15]. TolC is shared by several efflux systems, hence *E. coli* strains deficient in TolC are more sensitive to a wider variety of chemicals (e. g. detergents, drugs, bile salts, and organic solvents) [16,17].

With the dedication of many research groups, the structure and mechanism of drug efflux by the RND pumps have been brought to light. The first crystal structure of the pump component was determined for TolC by Koronakis et al. [18] in 2000. TolC is a trimer with an overall length of 140 Å with 40 Å in the β-barrel domain mainly composed of β strands, and 100 Å in the periplasmic domain mainly composed of α-helices. The periplasmic end of the TolC tunnel is sealed at the resting state, which likely opens by an allosteric protein–protein interaction mechanism [19]. In 2002, Murakami et al. first reported the crystal structure of AcrB, followed by the proposal of the functional rotation mechanism [20,21,22]. Later in 2006, Mikolosko and coworkers determined the crystal structure of AcrA. In contrast to the trimeric TolC and AcrB, AcrA forms a hexamer in the pump assembly [23]. The assembled pump structure was first proposed as the “deep interpenetration model”, which shows that AcrB and TolC have direct interactions with AcrA wrapped around on the outside to strengthen the interaction [24]. More recently, Wang et al. proposed a new model based on Cryo-EM studies, known as the “tip-to-tip model”. In this model, AcrA hairpins form a barrel-like conformation, contacting TolC in a tip-to-tip arrangement [25,26]. The recent determination of the complex structure first by cryo-EM, then by X-ray crystallography, confirmed the tip-to-tip model [7,19,20,27,28,29,30]. Energy does not seem to be required to assemble the AcrAB-TolC complex and AcrAB could interact with the TolC channel to form a AcrAB-TolC complex even in the absence of known substrate [31]. The dynamic process that leads to the formation of the complex is still elusive.

The dominant negative effect describes the phenomena in which an excess of a functionless mutant of a protein in the presence of its wild type counterpart, reduces the observed activity due to competition from the mutant for interaction with functional partners of the protein of interest. In the AcrAB-TolC complex, over-expression of functionless AcrB or AcrA mutant in wild type *E. coli* strains is expected to drastically reduce the assembly of functional efflux complex, and thus reduce the efflux activity and increase the sensitivity to substrate compounds. However, we tested the overexpression of several functionally defective mutants in the wild type *E. coli* strain, but did not observe the expected level of reduction. We speculate that the assembly of the AcrAB-TolC is a precisely controlled process involving delicate proof-reading procedures.

## 2. Results

### 2.1. AcrB Mutants Defective in Proton Transport

Several key residues have been identified in the AcrB transmembrane domain, forming the proton translocation pathway [20,30,32,33,34,35,36,37,38]. We have created single alanine replacement mutations at each of these sites to obtain mutants AcrB-D407A, AcrB-D408A, AcrB-K940A, AcrB-T978A, and AcrB-R971A [34,37]. The plasmid encoding of these mutants was first transformed into the BW25113∆*acrB* strain to examine their efflux activity (Table 1). As expected, the minimum inhibitory concentrations (MICs) of most examined substrates against the strains containing the mutants were the same as those against the strains without plasmids. The only mutant that displayed significant activity is T978A, which remained partially active. As a positive control, we showed that transformation with a plasmid encoding the wild type AcrB completely restored the efflux activity with MIC values similar to a wild type BW25113 strain. Next, we transformed these plasmids into the wild-type BW25113 strain and measured the MIC. We expected the AcrB mutants to compete with the genomic AcrB in binding and interaction with genomic AcrA and/or TolC, thus reduce the number of functional efflux complexes and subsequently the drug susceptibility. However, we observed a two-fold reduction of MIC value in some cases, and no reduction in others, which is consistent with an earlier study reporting the modest reduction of substrate susceptibility when AcrB-D407A was over-expressed in a wild -type *E. coli* strain (Table 1) [39].

**Figure 1:**
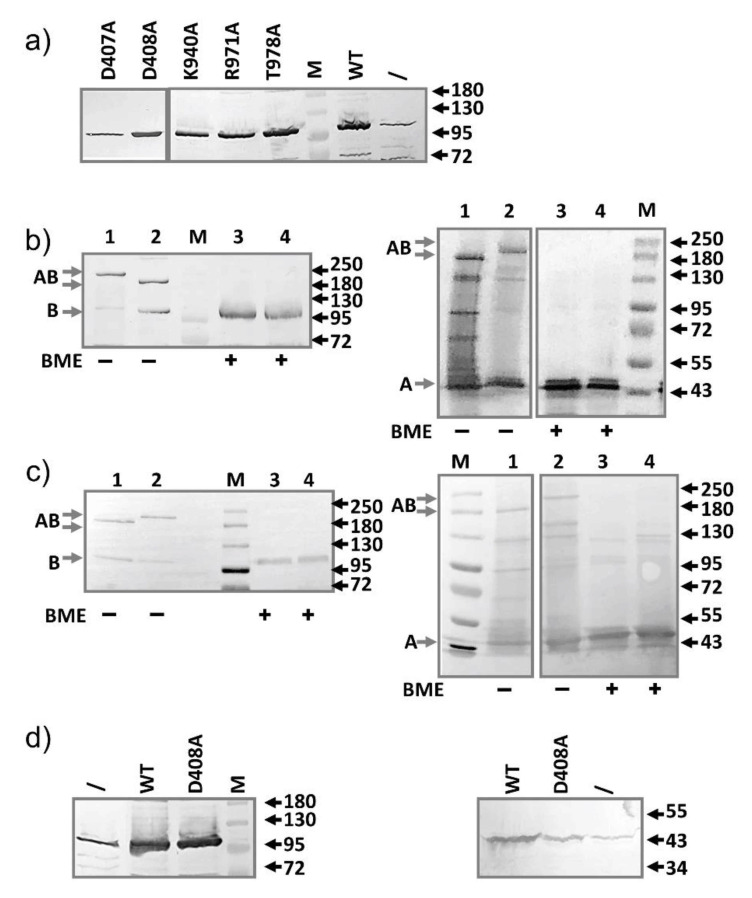
Characterization of mutants defective in the proton translocation pathway. (**a**) The point mutation did not affect expression level. Anti-AcrB Western blot analysis of the expression of all five mutants and the wild-type AcrB from plasmid transformed into BW25113. Sample prepared from plasmid-free BW25113 (\) was also prepared and loaded to serve as a control to highlight the difference in expression levels. (**b**) Anti-AcrB and Anti-AcrA Western blot analyses revealing the formation of disulfide bonded AcrA-AcrB complexes, which was reduced after incubation with BME. AcrA-P57C/AcrB-N191C (lane 1 and 3), AcrA-T217C/AcrB-S258C (lane 2 and 4). (**c**) Similar to b, with the additional D408A mutation introduced into the constructs. AcrA-P57C/AcrB-N191C/AcrB-D408A (lane 1 and 3), AcrA-T217C/AcrB-S258C/AcrB-D408A (lane 2 and 4). Molecular weight markers are labeled as “M” and the molecular weight of bands (kD) were indicated on the right. The expected bands for AcrA, AcrB, and disulfide bond linked AcrA-AcrB are marked on the left of the gels as A, B, and AB, respectively. (**d**) Anti-AcrB (left) and anti-AcrA (right) Western blot analysis of BW25113 expressing plasmid pBAD33-AcrAB (WT) or pBAD33-AcrAB-D408A (D408A). Samples prepared from BW25113 not containing plasmid was used as the control (/). For anti-AcrA Western blot, plasmid-containing samples were diluted 4-fold before being loaded into the gel.

We conducted the MIC assay under the basal expression condition without induction to avoid the potential artifact that may arise from over-expression. To examine how much of each mutant actually expressed under the basal condition, we prepared samples from BW25113 containing different plasmids, and compared the expression level from the plasmid to the level of genomic AcrB. Even without induction, under our experimental condition, the plasmid-encoded mutants were expressed at a much higher level (5–20 folds higher) compared to the level of the genomic AcrB (Figure 1a). This result indicates that the lack of impact on drug susceptibility is not due to the lack of expression. Even presented at a large excess, the mutants were not effective in disrupting the normal efflux activity.

Single mutations on the proton relay pathway do not significantly affect the overall structure of AcrB. The crystal structure of a couple of these mutants have been determined using X-ray crystallography [34,40]. All mutants form trimeric structures similar to those in the wild-type AcrB [34]. To examine if a mutant defective in proton translocation could still bind AcrA, we used two inter-subunit disulfide bonds as our yardsticks to probe the interaction between AcrA and AcrB. We first constructed a plasmid expressing both AcrA and AcrB (pBAD33-AcrAB), then introduced a pair of cysteines, one in AcrA and the other in AcrB: AcrA-P57C/AcrB-N191C, and AcrA-T217C/AcrB-S258C. These residues are predicted to be close to each other according to the “Disulfide by Design 2.0” (http://cptweb.cpt.wayne.edu/DbD2/ (accessed on 6 January 2019)) [41]. The formation of disulfide bond linked AcrA-AcrB complex was confirmed using anti-AcrA and anti-AcrB Western blot (Figure 1b). A high molecular weight complex could be detected in both blots, which disappeared upon incubation with β-mercaptoethanol (BME). The disulfide bond-linked species migrated slightly differently in the gel, likely due to differences in the conformations of the two complexes under the gel running condition. Mutation and disulfide bond formation did not significantly impair efflux activity, as revealed in the MIC measurement (Table 2). Next, we introduced the D408A mutation into both constructs to examine the effect of this additional mutation on the formation of disulfide bond linked AcrA-AcrB complex. If the D408A mutation had a significant impact on the interaction between AcrB and AcrA, we expect to see a reduction of the intensity of the high molecular weight AcrA-AcrB complex. As shown in Figure 1c, disulfide bond formation was to a similar level in both constructs, suggesting that the additional D408A mutation did not have a significant impact on AcrA-AcrB interaction.

To examine if the additional expression of AcrA from the same plasmid as the AcrB-D408A have any impact on the competition with the genomic AcrB, we introduced plasmid pBAD33-AcrAB-D408A into the wild type BW25113 strain and examined the MIC. Both AcrA and AcrB-D408A expressed at levels much higher than their genomic counterparts (Figure 1d), and yet, no dominant negative effect was observed (Table 2).

### 2.2. AcrB Mutants Defective in Substrate Binding

One feature of the AcrAB-TolC complex that has drawn much research interest is their ability to efflux a large array of substrates ranging broadly in molecular weight, charge, and hydrophobicity. Many mutations have been introduced in the substrate binding pocket in AcrB to probe their impact on the efflux of different substrates [30,42]. We chose three such mutants to include in this study, F610A, I278A, and F178A, since they were reported to have the most significant impact on efflux. First, plasmids containing single residue mutations at these sites were introduced into BW25113∆*acrB* to examine their activities (Table 3). While the F610A mutant is largely inactive, both F178A and I278A remained partially active, which is consistent with previous reports [43]. It is clear that in general, single point mutations introduced at the substrate binding site are not as detrimental as mutations introduced in the proton translocation pathway. This is reasonable when considering several residues collectively form a substrate binding site, while the proton translocation pathway is more linear.

Next, plasmids encoding these mutations were transformed into BW25113 to determine their impact on efflux activity. Similar to proton relay pathway mutants, the MIC values of the strain were not significantly affected (Table 3). The presence of AcrB mutants defective in substrate binding does not display the dominant negative phenotype either.

**Figure 2:**
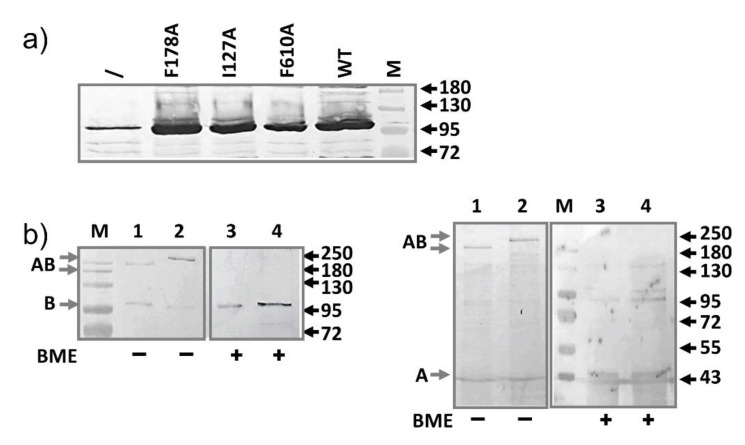
Characterization of AcrB mutants defective in substrate binding. (**a**) The point mutation did not affect expression level. Anti-AcrB Western blot analysis of basal expression of all three mutants and the wild type AcrB from plasmid transformed into BW25113. Sample prepared from plasmid-free BW25113 (\) was also prepared and loaded to serve as a control to highlight the difference in expression levels. (**b**) Anti-AcrB and Anti-AcrA Western blot analyses revealing the formation of disulfide bonded AcrA-AcrB complexes, which was reduced after incubation with BME. AcrA-P57C/AcrB-N191C, F610A is in lane 1 and 3, and AcrA-T217C/AcrB-S258C, F610A in lane 2 and 4. Molecular weight markers are labeled as “M” and the molecular weight of bands (kD) were indicated on the right. The expected bands for AcrA, AcrB, and disulfide bond linked AcrA-AcrB are marked on the left of the gels as A, B, and AB, respectively.

Next, we examined the expression of these mutants under the basal condition (Figure 2a). Similar as described above, the plasmid-encoded mutants were expressed at a much higher level compared to the level of the genomic AcrB, indicating that the lack of impact on MIC is not due to the lack of expression.

To determine if a mutation in the substrate binding pocket of AcrB (F610A) affects interaction between AcrB and AcrA, we used the disulfide bond pairs as described above and introduced an additional AcrB F610A mutation (Figure 2b). Similar as in Figure 1b, AcrA-AcrB complexes were observed, similar as in samples without the F610A mutation, suggesting that the AcrB F610A mutant still binds with AcrA.

### 2.3. AcrA Mutant Defective in TolC Interaction

AcrA is the periplasmic adaptor protein of the efflux system. While debate still exists concerning the conformation of AcrA in the final assembled pump, it was clear that several residues at the tip of its long α-helical hairpin loop play an important role in the interaction with TolC [44]. We created three such mutants, R128D, L132D, and S139D, and introduced plasmids encoding these mutants first into BW25113∆*acrA* to confirm that they are not active (Table 4). As expected, the mutants were largely inactive. The plasmids were then transformed into BW25113 and substrate susceptibility of the strains were measured. Similar to the AcrB mutants, we did not observe the dominant negative effect.

**Figure 3:**
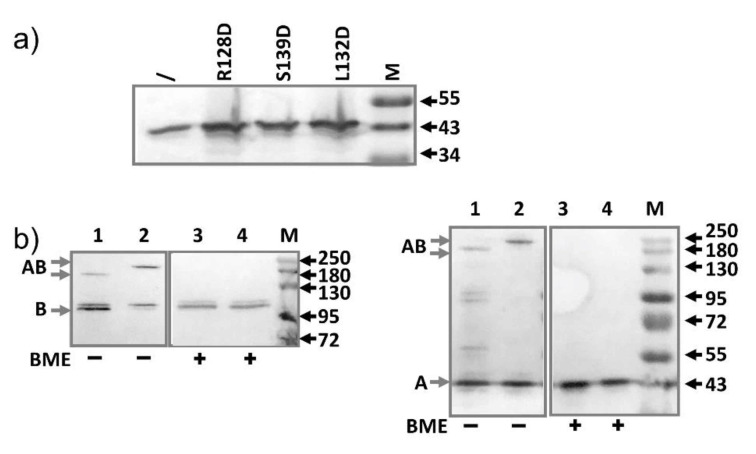
Characterization of AcrA mutants defective in TolC interaction. (**a**) Anti-AcrA Western blot analysis of samples prepared from BW25113 containing plasmids expressing the indicated AcrA mutant (diluted 4- folds). Sample prepared from plasmid-free BW25113 was also prepared and loaded without dilution (\) to serve as a control to highlight the difference in expression levels. (**b**) Anti-AcrB and Anti-AcrA Western blot analyses revealing the formation of disulfide bonded AcrA-AcrB complexes, which was reduced after incubation with BME. AcrA-P57C/AcrB-N191C, R128D (lane 1 and 3), AcrA-T217C/AcrB-S258C, R128D (lane 2 and 4). Molecular weight markers are labeled as “M” and the molecular weight of bands (kD) were indicated on the right. The expected bands for AcrA, AcrB, and disulfide bond linked AcrA-AcrB are marked on the left of the gels as A, B, and AB, respectively.

Next, we examined the expression of AcrA from the plasmids. As discussed above for AcrB mutants, we did not induce expression. Both the MIC study and the expression level detection experiments were performed under the basal expression condition. Expressions of the mutants (R128D, L132D, S139D) were higher than the expression level of the genomic AcrA (Figure 3a). To make the sample intensity comparable on the Western blot analysis, samples prepared from plasmid-containing strains were diluted 4-fold before being loaded into the gel. Thus, the expression levels of the mutant AcrA constructs were 10–20 folds higher than the expression level of the wild type AcrA from the genome. While these mutants had been studied for their impact on interaction with TolC, it was not clear if they would have an impact on binding with AcrB. We used the double Cys mutants as described above to probe the interaction between AcrA-R128D and AcrB (Figure 3b). This extra mutation did not seem to disrupt the interaction between AcrA and AcrB.

### 2.4. AcrA Mutant Defective in AcrA Assembly

**Figure 4:**
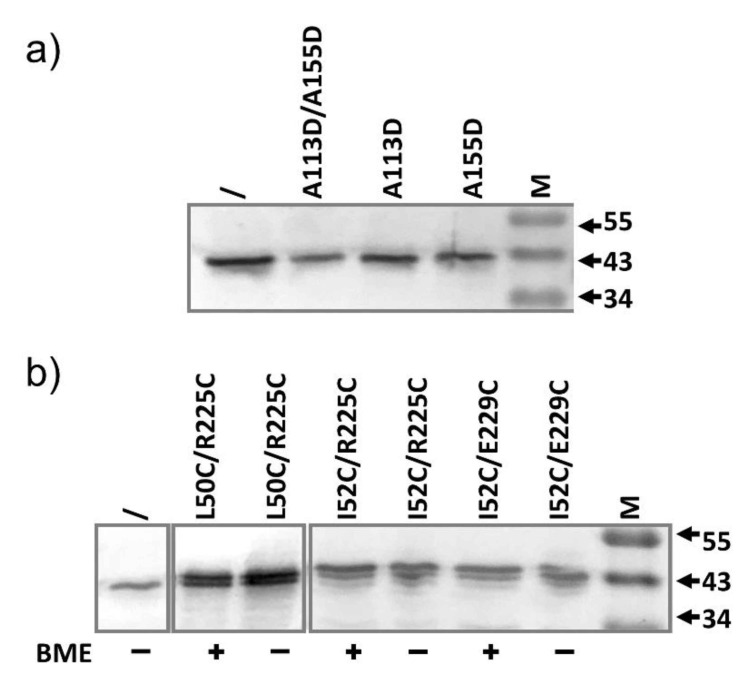
Anti-AcrA Western blot analysis of samples prepared from BW25113 containing plasmids expressing the indicated AcrA mutant (diluted 4-folds). Sample prepared from plasmid-free BW25113 was also prepared and loaded without dilution (\) to serve as a control to highlight the difference in expression levels. (**a**) AcrA mutants are expected to affect AcrA–AcrA interaction during pump assembly. (**b**) AcrA mutants forming intra-subunit disulfide bond to be trapped in an inactive conformation. Addition of BME did not lead to an observable change in mobility.

According to the cryo-EM structure of the complex, AcrA forms a hexameric barrel, with each subunit contributing a long helical hairpin. We speculate that mutations introduced at neighboring sites between the hairpins would disrupt the interaction between neighboring AcrA subunits, and thus disrupt the formation of the helical barrel. We chose two sites to introduce mutation, A113 and A155. They are located at the inter-subunit interface in the middle of the long hairpin. We created both single and double mutants containing A113D and A155D. Plasmids encoding these strains were first transformed into BW25113∆*acrA* to examine the impact of the mutation on the efflux activity (Table 4). Both mutations disrupted efflux, as revealed by a reduction of the MIC. The A155D mutation is more detrimental than A113D. Both A155D and the double mutations A113D/A155D were largely inactive. When transformed into BW25113, we still did not observe a significant reduction of MIC. The expression levels of the mutants were examined to confirm that mutants did express in excess compared to the level of the genomic AcrA under the experimental condition. As shown in Figure 4a, the expression of the mutants was similar or slightly higher than that of the genomic AcrA. We next used the inter-AcrA/AcrB disulfide bond pairs to examine the impact of A155D mutation on the interaction between AcrA and AcrB (data not shown). This additional mutation did not have a significant impact on the level of disulfide bond formation, indicating that it did not disrupt the interaction between AcrA and AcrB.

### 2.5. AcrA Mutant Defective in Conformational Change

We created three pairs of defective AcrA mutants that are trapped in a nonfunctional confirmation via a disulfide bond, L50C-R225C, I52C-R225C, and I52C-E229C [45]. Hazel et al. first created these mutants to examine their hypothesis that AcrA adopts two conformations, a *cis*-like conformation in which membrane proximal (MP) and α-helical domains point to the same direction, and a *trans*-like conformation in which they point to opposite directions. We created the corresponding single and double mutants and confirmed that the double mutants were largely inactive when transformed into BW25113∆*acrA* (Table 4), consistent with previous report. Yet, expression of these mutants in the BW25113 strain did not have a significant impact on the efflux of AcrAB-TolC substrates. We have also examined the expression of the mutants and confirmed that they were present in the cells at a higher level than the genomic AcrA (Figure 4b).

### 2.6. AcrA Mutants in a Strain Containing Anchor-Free AcrA

Finally, we examined the potential contribution of the lipid anchoring of AcrA on the stability of AcrA-AcrB interaction. Toward this goal, we engineered a BW25113 strain (BW25113*spmut*) in which the signal peptide of AcrA (residue 1–24 encoded in the *acrA* gene) in the genome was replaced with the signal peptide of OmpA. The OmpA signal peptide directs the secretion of the AcrA to the periplasm after synthesis. The lipid anchoring was abolished, but the activity of AcrA was not affected [13]. We reason that removal of the lipid anchoring might favor dissociation of AcrA from AcrB in the AcrA-AcrB complex, and thus providing an opportunity for the mutant AcrA, which is lipid-anchored, to compete more effectively for binding with AcrB. We transformed above-mentioned AcrA mutant into BW25113*spmut* and measured the MIC of the strains (Table 5). We still did not observe a significant drop in efflux activity.

### 2.7. Slow Dissociation of the AcrAB Complex

**Figure 5:**
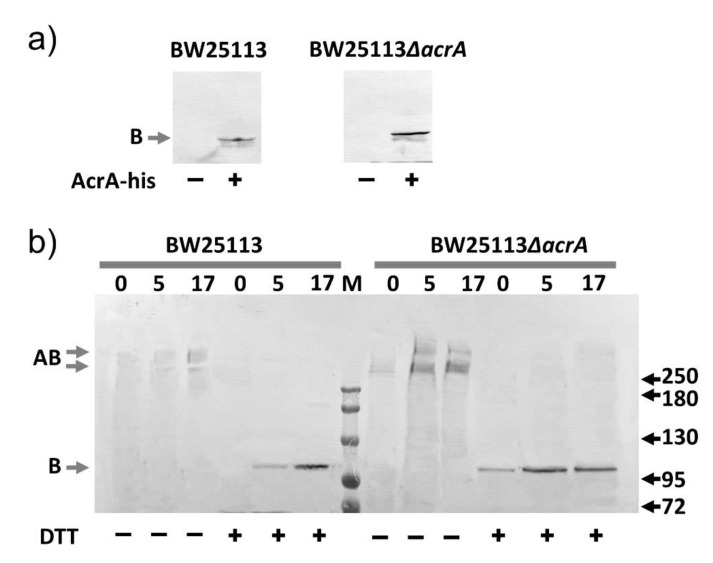
Co-purification of genomic AcrB with AcrA-his. (**a**) Anti-AcrB Western blot analyses of samples prepared from BW25113 or BW25113Δ*acrA* with or without plasmid-encoded AcrA-his. (**b**) Anti-AcrB Western blot analyses of samples collected after 0, 5, or 17 h of incubation following the induction of AcrA-his production in BW25113 or BW25113Δ*acrA* strains. Dithiobis-(succinimidyl proprionate) (DSP) crosslinking was performed to stabilize the AcrAB complex before protein purification. Reduction using dithiothreitol (DTT) breaks the disulfide bond in the linker of DSP and the complex into AcrA and AcrB subunits. Molecular weight markers are labeled as “M”, and the molecular weight of bands (kD) were indicated on the right. The expected bands for AcrB and DSP linked AcrA-AcrB are marked on the left of the gels as B and AB, respectively.

We speculate that the AcrAB complex, once formed, dissociates very slowly. To experimentally test this speculation, we introduced a plasmid encoding AcrA bearing a histag at the C-terminus (AcrA-his) into a wild-type and the corresponding *acrA* knockout strains. We first confirmed that under our experimental condition, the genomic AcrB could not be purified using metal affinity chromatography (Figure 5a). In the absence of the plasmid, no AcrB could be detected in the eluate in anti-AcrB Western blot. In contrast, when AcrA-his was introduced into the cells, AcrB could be detected in the eluates, indicating that it was co-purified through interaction with AcrA-his. Interestingly, the AcrB band intensity was higher in the eluate prepared from the *acrA* knockout strain, indicating more AcrB were co-purified. Next, we examined the time course of co-purification. The rationale is, in the wild-type strain, genomic AcrB and AcrA form stable complexes. If the dissociation is fast, the introduced AcrA-his will quickly compete with genomic AcrA to form a complex with AcrB, and in turn enable purification of AcrB through metal affinity chromatography. Otherwise, if the dissociation is slow, then it takes much longer for the competition to happen. We monitored the formation of AcrAB complex between the genomic AcrB and plasmid-expressed AcrA-his at three-time points, right after the induction period, and 5 and 17 h after induction (Figure 5b). We performed crosslinking right before protein extraction and purification to stabilize the complexes. As a control experiment, we also examined the formation of the AcrAB complex between the genomic AcrB and plasmid-expressed AcrA-his in an *acrA* gene knock-out strain. In this case, we do not expect competition from the genomic AcrA; thus complex should form faster. We found that the formation of the complex is plateaued much faster in the *acrA* knockout strain, as intensities of the eluates prepared from 5 and 17 h into incubation were very similar. In contrast, it took much longer for AcrB to interact with AcrA-his in the wild type strain, which is likely due to the requirement of an extra step of AcrAB dissociation between the genomic AcrA and AcrB.

## 3. Discussion

The dominant negative effect describes the situation in which the phenotype is dominated by the negative impact of the functionless mutant. The observation of the dominant negative effect has been used in many studies to investigate the mechanism of protein–protein interaction, including the identification of protein–protein interactions interface [46], determination of enzymatic activity related to oligomerization [47], and the effect of mutations in genetic disorders [48,49].

**Figure 6:**
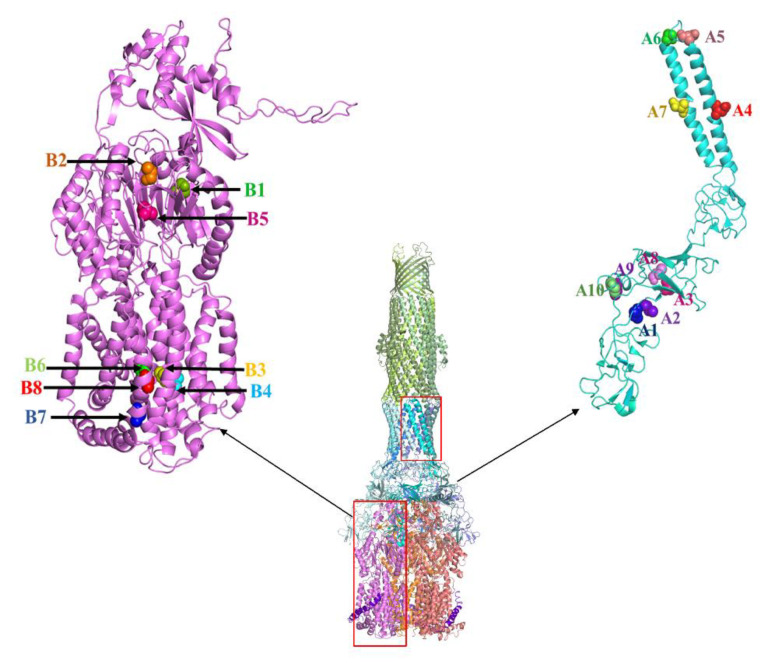
Structure of the AcrAB-TolC complex with the residues mutated in this study highlighted in AcrA and AcrB structures. AcrA mutations labeled as; A1-L50C, A2-I52C, A3-P57C, A4-A113D, A5-L132D, A6-S139D, A7-A155D, A8-T217C, A9-R225C, A10-E229C. AcrB mutations are labelled as; B1-F178A, B2-I278A, B3-D407A, B4-D408A, B5-F610A, B6-K940A, B7-R971A, B8-T978A. AcrAB crystal structure is created using pymol from 5N5G.pdb (https://pymol.org/2/ (accessed on 15 Marth 2021)).

In the process of AcrAB-TolC assembly, there are many steps where the incorporation of a functionless AcrA or AcrB mutant would negatively impact the efflux activity. First, all three proteins in the system are oligomers. AcrB and TolC are obligate timers, while AcrA is believed to exist as a dimer or trimer in the free form and assembles into a hexamer in the pump complex [13,50]. While AcrA and AcrB are believed to form a complex in the absence of substrate and efflux, TolC assembles with AcrAB during active efflux. When a functionless AcrA mutant is expressed in excess in a wild type *E. coli* cell containing genomic AcrA, we expect them to compete with their genomic counterpart to engage genomic AcrB, forming non-functional interactions to reduce the overall efflux activity. Similarly, we expect functionless AcrB mutant expressed from a plasmid to compete with genomic AcrB for genomic AcrA. In addition, we expect the competition for genomic TolC will further enhance the dominant negative effect. For this competition to occur, we chose mutants that are defective due to mechanisms not directly related to the interaction between AcrA and AcrB. The structure of the AcrAB-TolC complex and location of mutants mentioned in this study are shown in Figure 6. We determined the expression level of the mutants relative to their genomic counterpart. Using serial dilution and quantitative Western blot analysis, we found that the expression levels of the AcrB mutants were 10–20 folds of the level of the genomic AcrB, and the AcrA mutants were 2 to 20 folds of the level of the genomic AcrA. With this high level of excess, we expect to observe a strong dominant-negative effect if the mutants were actively involved in the pump assembly, competing for binding partners. We constructed five groups of AcrA and AcrB mutants, defective in different aspects. We observed that the effect of certain mutations was not always the same for different substrates. For example, T978A mutation in AcrB is detrimental to all substrates tested except for R6G, while F178A mutation in AcrB drastically reduced the MIC for ERY, but not as much for other substrates tested. This difference in mutation effects has been observed in many studies characterizing AcrA and AcrB mutants, for example, [23,42,45]. We speculate that this difference could be due to differences in the binding and interaction of specific substrates with the pump complex. The substrates vary drastically in their size, shape, structure, and charged state. As a result, the subgroup of residues that they interact with on their way to be transported are not likely to completely overlap. Therefore, point mutations introduced in AcrA and AcrB could have different impacts on specific substrates.

Mutants defective in the proton translocation pathway still form trimers [34] and interact properly with AcrA (Figure 1c). Then, was why no dominant negative effect observed? One possibility is that the genomic AcrA and AcrB are transcribed together, sharing the same mRNA. Hence, the newly produced AcrA and AcrB could be clustered as well. As a result, the genomic AcrA and AcrB form a AcrAB complex as soon as they are translated and inserted into the membrane (AcrB) or secreted into the periplasm with lipid anchoring (AcrA). Since the local concentration of the genomic proteins are high, they associate with each other with a much higher chance than associate with a plasmid-encoded partner. Another requirement for the observed activity is that the AcrAB complex, once formed, should be resistant to dissociation. Otherwise, the high concentration of AcrB mutant in the cell membrane would be effective in competing with genomic AcrB to form a nonfunctional AcrAB complex.

We further examined the impact of over-expressing both AcrA and AcrB, in which the entire sequence coding AcrA and AcrB were inserted in a plasmid. As a result, we expect them to form AcrA-AcrBD408A complex to compete with plasmid encoded AcrAB for genomic TolC. Yet, while the expression level of the complex was ~20 fold higher than that of the genomic AcrAB, no significant reduction of MIC was observed. It appears that TolC could differentiate the two complexes, even though the only difference between them is the single residue mutation down in the transmembrane domain of AcrB.

The second group of AcrB mutants examined are defective in substrate binding. These mutations are not expected to affect AcrB structure [42,51], nor do they impact the interaction between AcrA and AcrB (Figure 2b). However, we did not observe a significant reduction of MIC.

We also examined the effect of expressing AcrA mutants on the efflux activity. The first group of residues we tested are ones that impact AcrA interaction with TolC, as they are involved in the tip-to-tip interaction with TolC [44]. We confirmed that a representative mutant in this group, R128D, still binds to AcrB (Figure 3b). Similarly, we expect the expression of these mutants will lead to competition with genomic AcrA for genomic AcrB, and the formation of a nonfunctional complex. However, no reduction of MIC was observed. To further probe the interaction of AcrA and AcrB, we created a strain of *E. coli* that is genetically modified to replace the signal peptide of AcrA with the signal peptide of OmpA (BW25113*spmut*). The resultant AcrA can still be secreted into the periplasm and is fully functional, but the lipid anchoring is lacking [13]. With this free-floating AcrA, we expect the interaction between AcrA and AcrB to be weakened, which may increase the competitiveness of the plasmid encoded AcrA, which is lipid anchored. Yet, we did not observe a reduction of MIC when the functionless mutants were expressed in strain BW25113*spmut*.

The next group of AcrA mutants contains changes at the inter-subunit interface of AcrA to disrupt formation of the functional hexametric ring. While the A155D single mutation was enough to completely abolish activity, expression of the protein in BW25113 did not lead to a reduction of MIC. Finally, we examine a group of AcrA that forms disulfide bond locked conformation that is functionally incompatible. Again, similar as other groups, over-expression of these mutants did not have a significant impact on MIC.

In conclusion, we examined the effect of plasmid-encoded AcrA and AcrB mutants in wild type *E. coli* cells, to probe the potential disruption of normal AcrA-AcrB-TolC assembly in the presence of excess mutants of AcrA or AcrB. To our surprise, none of the five groups of mutants showed the so-called “dominant negative” effect. This observation indicates that the RND pump assembly process in Gram-negative bacteria is a precisely controlled process that prevents the formation of functionless complex. An alternate explanation is the possibility that efflux depends on only a very small population of AcrAB-TolC pumps active at a given moment, as the population of active AcrAB-TolC pumps was not detectable in situ in *E. coli* [19]. If the majority of AcrAB and TolC in the cells are idle, then the effect of over-expressing functionless mutant would be greatly limited. In addition, our results suggest that dissociation kinetics of the AcrAB complex is very slow. Once formed, the complex remains bound and does not dissociate easily.

## 4. Materials and Methods

### 4.1. Bacterial Strains, Plasmids, and Growth Conditions

*Escherichia coli* BW25113 and BW25113∆*acrB* were obtained from Yale *E. coli* genetic resources. BW25113∆*acrAB*, BW25113∆*acrA* were constructed using the *E. coli* gene deletion kit (Cat #K006, Gene Bridge) following the manufacturer’s protocol. Plasmids pBAD33-AcrB and pBAD18-AcrA were created in our previous study [52]. To create the plasmid pBAD33-AcrAB, *acrAB* gene was amplified from genomic DNA of BW25113 and cloned into pBAD33 by following the fast cloning method described previously by Li et al. [53]. Plasmids pBAD33-AcrB, pBAD33-AcrAB, and pBAD18-AcrA were used as the templates to create respective mutations discussed below. Mutations were introduced using the Quikchange site-directed mutagenesis kit following manufacturer’s instructions (Agilent, Santa Clara, CA, USA). BW25113spmut, in which the signal peptide of AcrA was exchanged with the signal peptide of outer membrane protein OmpA, was created using the CRISPR-Cas9 system by following the published protocol [54]. Plasmids pTargetF and pCas were a gift from Shen Yang (Addgene plasmid #62226 and #62225) [54]. Bacteria were cultured at 37 °C with shaking at 250 rpm in Luria broth (LB) media unless otherwise noted.

### 4.2. Drug Susceptibility Assay

The minimum inhibitory concentration (MIC) was determined for erythromycin, novobiocin, ethidium bromide (EtBr), rhodamine 6G (R6G), nalidixic acid, and tetraphenylphosphonium chloride (TPP) following the CLSI guidelines [55]. Briefly, overnight cultures of the indicated strain were diluted to a final concentration of 10^5^ CFU/mL in fresh Muller Hinton Broth 2 (cation adjusted) media (Millipore Sigma, St. Louis, MO, USA) in a 48 well microtiter plate containing the indicated compounds at two-fold serial dilutions. Plates were incubated at 37 °C with shaking at 160 rpm for 17 h; the absorbance at 600 nm (OD600) were measured to identify the lowest concentrations with no observable cell growth. All experiments were repeated at least three times.

### 4.3. Protein Expression, SDS-PAGE and Western Blot Analysis

For expression test, 5 mL of cells were cultured overnight at 37 °C with shaking at 250 rpm. The next morning, the cell was inoculated with a 100-fold dilution into a 5 mL fresh LB media supplemented with antibiotics and grow until ~OD600 1.0. Cells were pelleted and resuspended in 1 mL phosphate buffer containing phenylmethylsulphonyl fluoride (PMSF) (1:1000 dilution of a saturated ethanol solution) and sonicated for 1 min followed by centrifugation for 10 min at 15,000 rpm. The supernatant was removed, and cell pellets were resuspended in 0.1 mL PBS containing 2% Triton-X100. The samples were incubated at room temperature with shaking for 45 min and centrifuged again for 10 min. The supernatant was used for SDS-PAGE and Western blot analysis. For studies of disulfide bond formation, iodoacetamide (IAM) was added to a final concentration of 20 mM in all buffers. To reduce disulfide bond, β-mercaptoethanol (BME) was added to a final concentration of 2% followed by incubation at room temperature for 30 min.

For AcrAB dissociation experiment, BW25113 or BW25113Δ*acrA* containing plasmid pBAD18-AcrA was cultured to the log phage (OD600 0.8) and arabinose was added to a final concentration of 0.2% (*w*/*v*) to induce the expression of AcrA for 50 min. The cells were then pelleted, washed, and resuspended with fresh LB. An aliquot of cell culture was collected, pelleted, and stored at −20 °C. The rest of the cell culture was returned to the shaker and cultured for 5 h, and another aliquot of sample was collected, pelleted, and stored at −20 °C. The last sample was collected at 17 h. OD600 of the samples were measured and used to adjust the sample volume collected to ensure that the same number of cells were used for each time point. All pellets were resuspended and sonicated to lyse the cells. After centrifugation, the pellet was extracted using PBS + 2% Triton for 2 h. The mixtures were centrifuged again and the supernatants were incubated with Ni beads for 40 min, followed by washing with the same buffer supplemented with 50 mM imidazole, and finally eluted with the same buffer supplemented with 500 mM imidazole. For DSP crosslinking experiments, the cell pellet was washed and then resuspended in PBS buffer. DSP was added to a final concentration of 1 mM, and the mixture was incubated at room temperature for 30 min. To stop the reaction, a Tris-Cl buffer (pH 8.0) was added to a final concentration of 20 mM. Cells were then pelleted, and proteins were purified similarly as described above. To break the disulfide bond in DSP, DTT was added to the sample to a final concentration of 10 mM.

For Western blot analysis, after transferring to the PVDF membrane, protein bands were detected using an Anti-AcrB polyclonal (Rabbit) antibody raised to recognize a C-terminal peptide corresponding to residues number 1036–1045 [39] or Anti-AcrA antibody, respectively. The membrane was then washed and incubated with an alkaline phosphatase conjugated goat anti-rabbit secondary antibody. The BCIP/NBT (5-bromo-4-chloro-3’-indolyphosphate and nitro-blue tetrazolium) solution was used to stain the membranes. All experiments were repeated at least three times.

## Figures and Tables

**Table 1:** MIC values (µg/mL) of BW25113 or BW25113∆*acrB* strains containing the indicated plasmid encoding AcrB mutants defective in proton translocation pathway.

Substrate ^1^	NOV	ERY	TPP	EtBr	R6G	NA
BW25113∆*acrB* containing						
/	4	4	4	8	8	1
WT	128	64	256	128	256	4
D407A	4	4	4	8	8	ND
D408A	4	4	4	8	8	ND
K940A	8	4	4	8	8	ND
R971A	8	4	4	8	16	ND
T978A	8	8	16	32	128	ND
BW25113 containing						
/	256	64	1024	512	1024	4
WT	512	128	1024	512	1024	4
D407A	128	64	1024	256	512	4
D408A	128	64	1024	512	1024	4
K940A	256	64	1024	512	1024	4
R971A	128	32	1024	512	1024	4
T978A	256	64	1024	512	1024	2

^1^ NOV, novobiocin. ERY erythromycin, TPP tetraphenylphosphonium, EtBr ethidium bromide. R6G, rhodamine 6G, NA, nalidixic acid. ND, not determined.

**Table 2:** MIC values (µg/mL) of BW25113 and BW25113∆*acrAB* strains containing the indicated plasmid encoding gene for both AcrA and AcrB.

Substrate	NOV	ERY	TPP	EtBr	R6G
BW25113∆*acrAB* containing					
/	4	4	8	4	16
WT	32	32	128	128	32
AcrA/AcrB-D408A	4	2	8	8	8
AcrAP57C/AcrBN191C	32	16	64	64	32
AcrA-T217C/AcrB-S258C	64	16	128	128	32
BW25113 containing					
/	256	32	1024	512	512
AcrAB-WT	256	64	1024	512	1024
AcrAB-D408A	256	32	1024	512	512

**Table 3:** MIC values (µg/mL) of BW25113 and BW25113∆*acrB* strains containing the indicated plasmid encoding AcrB mutants defective in substrate binding.

Substrate	NOV	ERY	TPP	EtBr	R6G	NA
BW25113∆*acrB* containing						
/	4	4	4	8	8	1
WT	128	64	256	128	256	4
F610A	8	8	64	32	128	ND
F178A	64	8	128	128	256	ND
I278A	32	32	128	128	128	ND
BW25113 containing						
/	256	64	1024	512	1024	4
WT	512	128	1024	512	1024	4
F610A	512	64	1024	512	1024	2
F178A	512	64	1024	512	1024	4
I278A	512	64	1024	512	1024	4

**Table 4:** MIC values (µg/mL) of BW25113 and BW25113∆*acrA* strains containing the indicated plasmid encoding AcrA mutants.

Substrate	NOV	ERY	TPP	R6G	NA
BW25113∆*acrA* containing					
/	4–8	2	4	4	1
WT	64	32	256	128	4
L132D	8	2	4	4	ND
R128D	4	2	4	4	ND
S139D	4	2	8–16	4	ND
A113D	64	4	32	16	ND
A155D	8	4	8	4	ND
A113D, A155D	8	2	8	4	ND
L50C	32	8	32	32	ND
I52C	32	4	16	32	ND
E229C	32	4	32	16	ND
R225C	16	8	64	16	ND
L50C, R225C	4	8	8	4	ND
I52C, R225C	4	4	8	8	ND
I52C, E229C	4	2	4	4	ND
BW25113 containing					
/	512	64	1024	512	4
R128D	512	64	1024	512	4
L132D	512	64	1024	512	4
S139D	256	64	1024	512	4
A113D	512	64	1024	512	4
A155D	512	64	1024	512	4
A113D, A155D	512	64	1024	512	4
L50C	512	64	1024	512	4
I52C	256	64	1024	512	4
E229C	512	64	1024	256	4
R225C	512	64	1024	512	4
L50C, R225C	512	64	1024	256	4
I52C, R225C	512	128	1024	512	4
I52C, E229C	512	128	1024	512	4

**Table 5:** MIC values (µg/mL) of BW25113*spmut* strain containing the indicated plasmid encoding AcrA mutants.

Substrate	NOV	ERY	TPP	R6G
/	512	128	2048	1024
R128D	512	128	2048	1024
L132D	512	128	2048	1024
S139D	512	128	2048	512
A113D	512	128	2048	512
A155D	512	128	2048	512
A113D, A155D	512	128	2048	512
L50C, R225C	512	128	2048	512
I52C, R225C	512	128	2048	1024
I52C, E229C	512	128	2048	1024

## Data Availability

The data presented in this study are available in the article.
